# Meglumine Exerts Protective Effects against Features of Metabolic Syndrome and Type II Diabetes

**DOI:** 10.1371/journal.pone.0090031

**Published:** 2014-02-27

**Authors:** Arturo Bravo-Nuevo, Alice Marcy, Minzhou Huang, Frank Kappler, Jennifer Mulgrew, Lisa Laury-Kleintop, Melvin Reichman, Annette Tobia, George C. Prendergast

**Affiliations:** 1 Lankenau Institute for Medical Research (LIMR), Wynnewood, Pennsylvania, United States of America; 2 Dynamis Pharmaceuticals Co. Inc., Jenkintown, Pennsylvania, United States of America; 3 LIMR Chemical Genomics Center Inc., Wynnewood, Pennsylvania, United States of America; Virginia Commonwealth University, United States of America

## Abstract

Metabolic syndrome, diabetes and diabetes complications pose a growing medical challenge worldwide, accentuating the need of safe and effective strategies for their clinical management. Here we present preclinical evidence that the sorbitol derivative meglumine (N-methyl-D-glucamine) can safely protect against several features of metabolic syndrome and diabetes, as well as elicit enhancement in muscle stamina. Meglumine is a compound routinely used as an approved excipient to improve drug absorption that has not been ascribed any direct biological effects *in vivo*. Normal mice (SV129) administered 18 mM meglumine orally for six weeks did not display any gastrointestinal or other observable adverse effects, but had a marked effect on enhancing muscle stamina and at longer times in limiting weight gain. In the established KK.Cg-Ay/J model of non-insulin dependent diabetes, oral administration of meglumine significantly improved glycemic control and significantly lowered levels of plasma and liver triglycerides. Compared to untreated control animals, meglumine reduced apparent diabetic nephropathy. Sorbitol can improve blood glucose uptake by liver and muscle in a manner associated with upregulation of the AMPK-related enzyme SNARK, but with undesirable gastrointestinal side effects not seen with meglumine. In murine myoblasts, we found that meglumine increased steady-state SNARK levels in a dose-dependent manner more potently than sorbitol. Taken together, these findings provide support for the clinical evaluation of meglumine as a low-cost, safe supplement offering the potential to improve muscle function, limit metabolic syndrome and reduce diabetic complications.

## Introduction

Physical exercise can help control glucose levels in individuals with metabolic syndrome or type II diabetes, triggering two different pathways of glucose uptake in skeletal muscle. One pathway involves well-established mechanisms controlled by insulin [1], [2], whereas the other involves little understood muscle contraction-related mechanisms that are independent of insulin [2], [3]. In recent work, muscle contraction has been reported to increase the activity of the AMPK-related enzyme SNARK (also known as NUAK2/ARK5), a kinase related to the master regulatory kinase AMPK that responds to metabolic stress [4], [5]. Mice that are genetically heterozygous for SNARK exhibit poor glycemic control, hyperinsulinemia, elevated plasma and liver triglycerides along with an increased body mass [6], [7]. Sorbitol is a slowly absorbed sugar alcohol derived from glucose and found in certain fruits [8]. It stimulates SNARK activity and increases glucose uptake by muscle cells [6]. Sorbitol has been explored as a supplement to increase glucose uptake in diabetic individuals, but its clinical use is impractical because its oral administration causes gastrointestinal distress and diarrhea[9]–[11]. Moreover, under diabetic conditions, accumulation of sorbitol in certain tissues, where it is only slowly cleared by the polyol pathway, may promote diabetic complications including atherosclerosis, cataracts, nerve damage and retinopathy [12].

Meglumine (N-methyl-D-glucamine) is a poorly metabolized [13] derivative of sorbitol that has regulatory acceptance as a benign excipient for drug formulation to increase aqueous solubility of lipophilic drugs and improve their absorption. Meglumine synthesis is simple and inexpensive, and, in several decades of clinical use, it has never been ascribed either detrimental or beneficial effects in patients or *in vivo* animal studies. *In vitro* studies have shown that meglumine can reduce oxidative damage in human fibroblasts cultured in a 3-deoxyglucosone (3DG) rich environment and increase fibroblast migration [14], [15]. Based on its structural similarity with sorbitol, we investigated whether meglumine may stimulate SNARK levels and exert muscle-associated protective effects against metabolic syndrome or diabetes-associated conditions in a preclinical mouse model of insulin-resistant diabetes.

## Materials and Methods

### Ethics Statement

This study was carried out in strict accordance with the recommendations in the Guide for the Care and Use of Laboratory Animals of the National Institutes of Health. The protocol was approved by The Lankenau Institute for Medical Research Institutional Animal Care and Use Committee approved (Permit Number: A11-2997).

### Animals

KK.Cg-Ay/J mice develop type 2 diabetes by 8 wk of age as characterized by onset of hyperglycemia, hyperinsulinemia, glucose intolerance and obesity [16]. 4–6 week old KK.Cg-Ay/J mice (n = 20 per group) were given water *ad libitum* with or without 18 mM meglumine hydrochloride (salt prepared from commercial meglumine obtained from Sigma, St. Louis, MO), kept in 12 hr night/day light cycles and fed *ad libitum*. Equal numbers of female and male animals were used.

### Glucose Measurement

Diabetes was confirmed in KK.Cg-Ay/J mice by blood glucose measurement using a “One Touch” blood glucose monitoring system (Lifescan, Milpitas, CA). Animals were considered hyperglycemic when, after a 4 hr fast, their blood glucose levels were above 250 mg/dl.

### Mesh Grip Test

Mice (SV129) (n = 15 per group) were treated with or without meglumine (18 mM in drinking water), for at least 30 days were placed on a mesh wire cage 50 cm above a cage filled with soft bedding. After adjustment to this new environment for 3–5 seconds, the cage was slowly inverted. Before the cage was inverted, the investigator visually confirmed that the animal was gripping the wire mesh on the cage with all limbs. After cage inversion, the grip time on the mesh wire cage before falling was recorded. This test was repeated 3 times for all mice with a 24 hr resting period between tests. The mean time mice held their grip on the mesh cage, while inverted, was employed as simple indicator of muscle strength [17].

### Nephropathy Analysis

Kidneys were examined from KK.Cg-Ay/J animals that had been hyperglycemic for 7 months. Renal slices were fixed in 4% paraformaldehyde, embedded in paraffin and deparaffinized in xylene before preparation of 4 µm sections and staining with hematoxylin-eosin, periodic acid Schiff (PAS) and Masson trichrome. The pathologist who scored the extent of renal injury was blind to the treatment groups. The score was based on morphometric analysis of the glomerular disease and interstitial fibrosis. A rating scale from 0 to 4 was used with 0 meaning normal appearance and 4 meaning very severe lesions.

### Glucose Challenge

After animals had fasted for 4 hrs, their basal glucose level was measured using a “One Touch” blood glucose monitoring system (Lifescan). They were then injected intraperitoneally (IP) with 1 g/Kg body weight of 10 mg/ml D-glucose in PBS. Blood glucose levels were measured at 15, 30, 60 and 120 minutes following injection.

### Lipid Analysis

Triglyceride levels were measured in mice using colorimetric assays. For this, lipids were extracted, from serum and liver, as described by Naik et al [18]. Briefly, after extraction and evaporation, the lipid layer was resuspended in 0.25 ml 1% Triton X-100. Triglyceride concentrations were then determined using the L-Type Triglyceride M enzymatic based assay kit (Wako Chemicals, Richmond VA). Results for liver tissue were expressed as microgram triglycerides per mg tissue.

### Western Analysis

C2C12 mouse myoblasts were cultured in Dulbecco’s modified Eagle medium (DMEM) (Mediatech, Manassas VA) containing 10% fetal bovine serum (Hyclone, Logan, UT), and maintained at 37°C in a humidified 5% CO_2_ atmosphere. Cells treated with or without meglumine at the concentrations and for the times indicated were harvested by scraping, washing twice in PBS and lysing in RIPA buffer containing protease and phosphatase inhibitors. Equal protein for each sample (typically 50 µg/lane) was separated by SDS–PAGE and transferred to Immobilon-P membranes (Millipore, Billerica MA). Blots were incubated with primary antibodies as recommended by the vendor (dilution 1∶1000) and were detected with HRP conjugated secondary antibodies using the enhanced chemilumescent reagents (Pierce, Rockford IL) according to the manufacturer’s instructions. Primary antibodies used were for SNARK (also known as NUAK2), MYPT1, phospho-MYPT1 (Ser507), phospho-MYPT1 (Ser668), phospho-MYPT1 (Thr696), (Cell Signaling Technology, Beverly, MA), Glut1 (Abcam, Cambridge, MA), AMPK (Millipore, Temecula, CA) and ß-actin (Santa Cruz Biotechnology, Santa Cruz, CA).

### Statistical Analysis

Values shown are means ± standard error of the means (SE). The significance of the differences between the two groups was analyzed by a student’s t test. P values of 0.05 or less were defined as statistically significant.

## Results

### Meglumine Stimulates SNARK in Myoblasts

**Figure 1:**
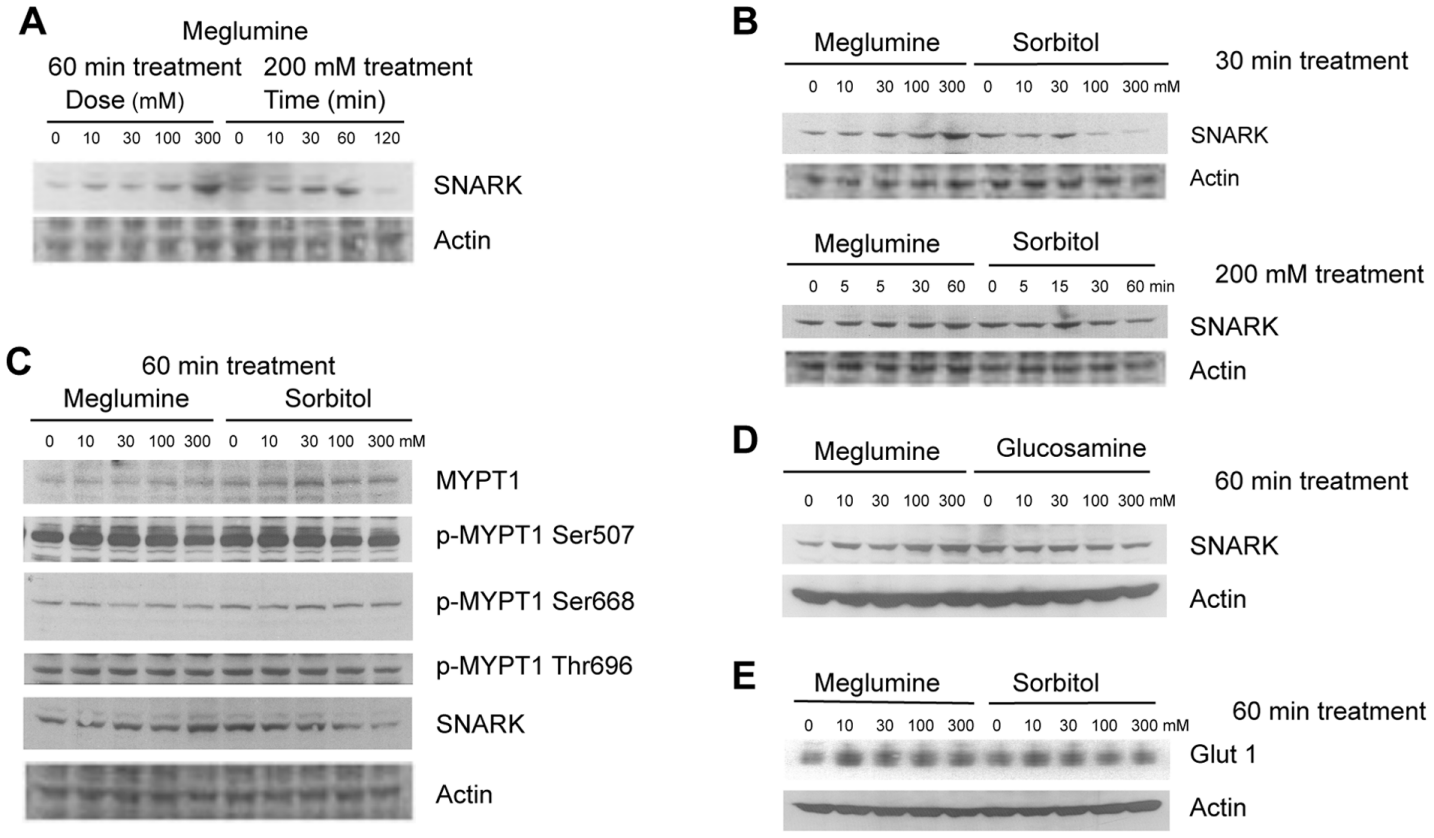
Meglumine increases SNARK levels in myoblasts. Panels show the results of Western analyses of total protein isolated from C2C12 murine myoblasts treated with meglumine, sorbitol or glucosamine at various concentrations and times. Actin levels were used as a control for equal protein loading in the gel lanes. A: Cells were treated with varying amounts of meglumine for 60 minutes (left side) or 200 mM meglumine for varying times (right side). B: Myoblasts were treated with various levels of meglumine or sorbitol for 30 min (left panel) or with 200 mM meglumine or sorbitol for various times (right panel). C: Cells were treated with varying amounts of meglumine or sorbitol for 60 minutes before analysis of the levels of SNARK or total or phospho-specific levels of its substrate MYPT1 phosphorylated at Ser507, Ser668 or Thr696. D: Cells were treated with varying amounts of meglumine or glucosamine for 60 minutes before analysis of SNARK levels.

Sorbitol-induced glucose uptake in muscle requires SNARK stimulation [6]. Given the close structural relationship of meglumine to sorbitol we asked whether the former might also stimulate SNARK. Murine C2C12 myoblasts were treated with sorbitol or meglumine for different times and concentrations. SNARK expression levels were determined by Western analysis. Steady-state levels of SNARK protein increased in a dose-dependent manner after 60 min of treatment with meglumine (Fig. 1A). This effect reached a plateau at 30 minutes of such that further increasing the time or concentration of meglumine did not further increase SNARK expression levels. The stimulatory effect of meglumine on SNARK levels was more potent and longer lasting than that produced by sorbitol (Fig. 1B). The increase in SNARK expression induced by meglumine was associated with an increase in phosphorylation of Ser507 on myosin phosphatase target subunit 1 (MYPT1) (Fig. 1C), a SNARK substrate [19] that regulates myosin light chain kinase and reinforces its effects on actin stress fiber formation and muscle contraction by the Rho kinase ROCK. In contrast, MYPT1 Ser668 or Thr696, which are also phosphorylated by ROCK appeared to be unaffected (Fig. 1C). To further establish the specificity of meglumine in affecting SNARK levels, we compared the effects of the amino sugar glucosamine (2-amino-2-deoxy-glucose), a structurally related nontoxic sugar. In contrast to meglumine, glucosamine did not stimulate SNARK expression (Fig. 1D). Finally, both meglumine and sorbitol caused an increase in the expression levels of glucose transporter Glut1, peaking at 10 mM treatment (Fig. 1E). Taken together, these observations prompted us to examine whether meglumine might affect muscle function or blood levels of glucose *in vivo*.

### Meglumine Lacks Diarrheal Effects but Increases Muscle Stamina and Limits Weight Gain in a Manner Associated with Steady-state Elevation of SNARK in Muscle

SNARK expression relates directly to muscle contraction and the activity of the Glut1 glucose transporter in muscle cells [6]. Since meglumine elevated SNARK expression in mouse C2C12 myoblasts, we investigated its effects in normal mice administered the compound at 18 mM in drinking water. This concentration was chosen from previous pilot studies based on pharmacokinetic analyses in rodents [13]. Starting an the age of 6 weeks, mice that received meglumine orally in their drinking water for a period of 12 weeks did not exhibit any change in their water consumption compared to control animals (Fig. S1). Further, meglumine-treated animals exhibited no signs of diarrhea (unpublished observations), which is the primary gastrointestinal side-effect produced by oral administration of sorbitol which prevents its clinical use.

**Figure 2:**
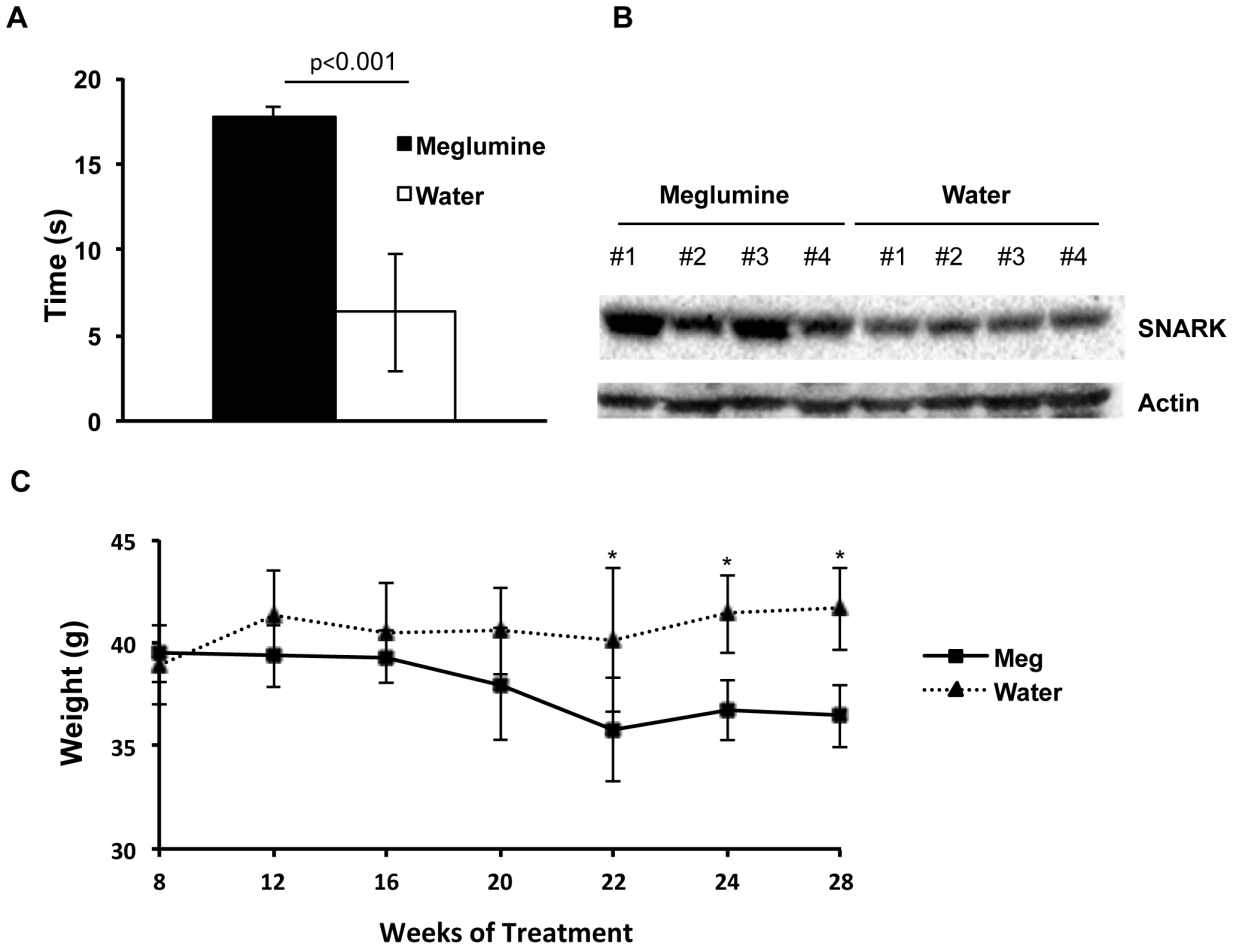
Positive effect of meglumine on skeletal muscle strength and weight gain. A: Control and meglumine-treated normoglycemic mice were subjected to a ‘hang test’ to measure strength. Meglumine treated mice (**black bar**) performed significantly better than control-treated animals, with the former group averaging 17.8 seconds of hanging time and the control group averaging 6.3 seconds. Values are mean ± SE with n = 15 for each group. P<0.001. B: Effect of meglumine in SNARK expression in skeletal muscle. Western analysis was conducted on total protein from tissue extracts prepared from dissected thigh muscle of animals euthanized at the experimental endpoint. C: Long term treatment with meglumine limits weight gain. Animals were weighed at the times indicated starting 8 weeks after initiation of 18 mM meglumine in drinking water. Asterisk designates P<0.05.

Since meglumine elevated SNARK in myoblasts, and SNARK has been suggested to cooperate with Rho/ROCK signaling to promote actin fiber formation and muscle contraction [19], [20], we reasoned that meglumine treatment might provide a benefit to muscle stamina. To explore this notion, we compared the length of time that control or meglumine-treated mice could grasp an inverted metal grid. This test was performed on each mouse once a day for several days, to permit sufficient rest between trials. We observed that meglumine-treated mice could suspend themselves above the cage significantly longer (17.8 sec) than mice in the untreated control group (6.3 sec) (Fig. 2A). This observation supported the notion that meglumine may affect muscle function in vivo. Since meglumine could elevate levels of SNARK in murine myocytes in vitro, we evaluated the levels of SNARK in vivo in skeletal muscle obtained from the thigh of mice euthanized at the experimental endpoint. Western analysis from four subjects in each group revealed variable but consistent upregulation in the steady-state levels of SNARK in the thigh muscle from meglumine-treated mice (Fig. 2B). While we did not explore stamina at longer treatment times, starting at 22 weeks we observed a significant trend toward lower weight in meglumine-treated mice compared to control mice receiving plain water (Fig. 2C). Together, these observations provided in vivo support for the hypothesis that meglumine may favorably influence muscle function and perhaps overall metabolic physiology at some level.

### Meglumine Improves Glycemic Control and Reduces Plasma Triglycerides in Diabetic Mice

Previously published studies have shown that SNARK heterozygote mice exhibit increased body weight, poor glycemic control, accumulated fat in liver and increased triglycerides in serum [6]. Based on the ability of meglumine to affect SNARK levels and muscle stamina, we assessed the compound’s effects on these parameters in KK.Cg-Ay/J mice, an established model of type II diabetes where hyperglycemia and insulin resistance develop by 2 months of age accompanied by nephropathy, fat accumulation in liver and increased serum triglycerides [16]. To assess long-term effects, mice were treated for 7 months with 18 mM of meglumine in drinking water as before and compared with an untreated control group. The KK.Cg-Ay/J model is not ideal to assess survival, but at this endpoint we observed no significant difference in the numbers of animals in each cohort.

**Figure 3:**
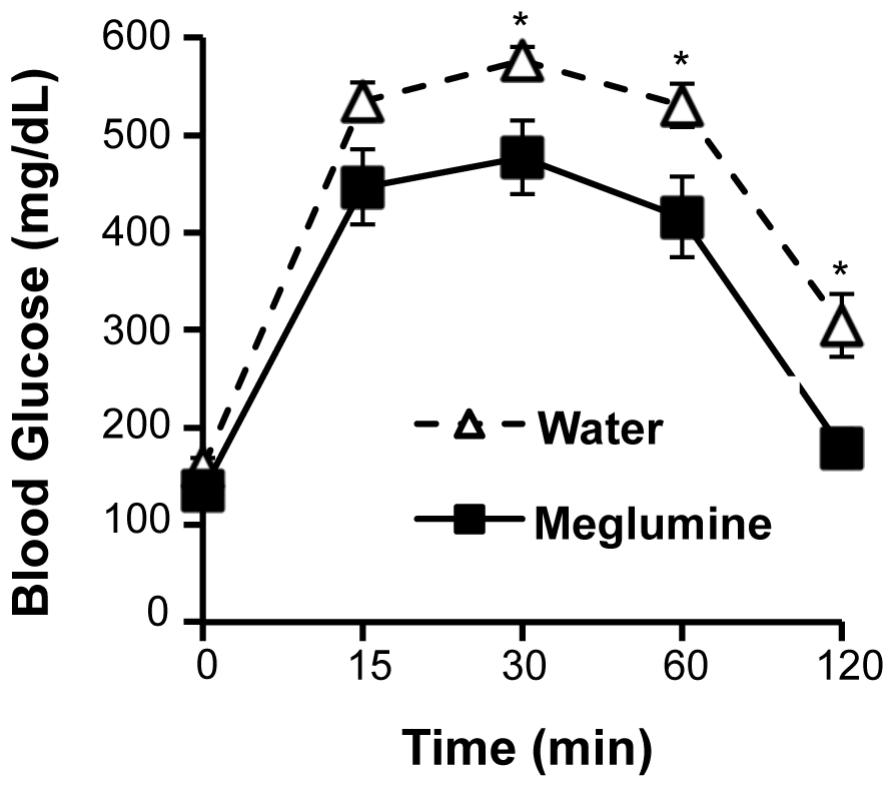
Meglumine improves glucose tolerance. Control (**dashed line**, open triangles) or meglumine-treated KK.Cg-Ay/J mice (**solid line,** closed squares) were injected i.p. with glucose (1 g/kg) and blood glucose was measured after 15, 30, 60 and 120 minutes. Values are mean ± SE shown in the error bar; n = 8 for each group. Asterisk designates P<0.05.

**Figure 4:**
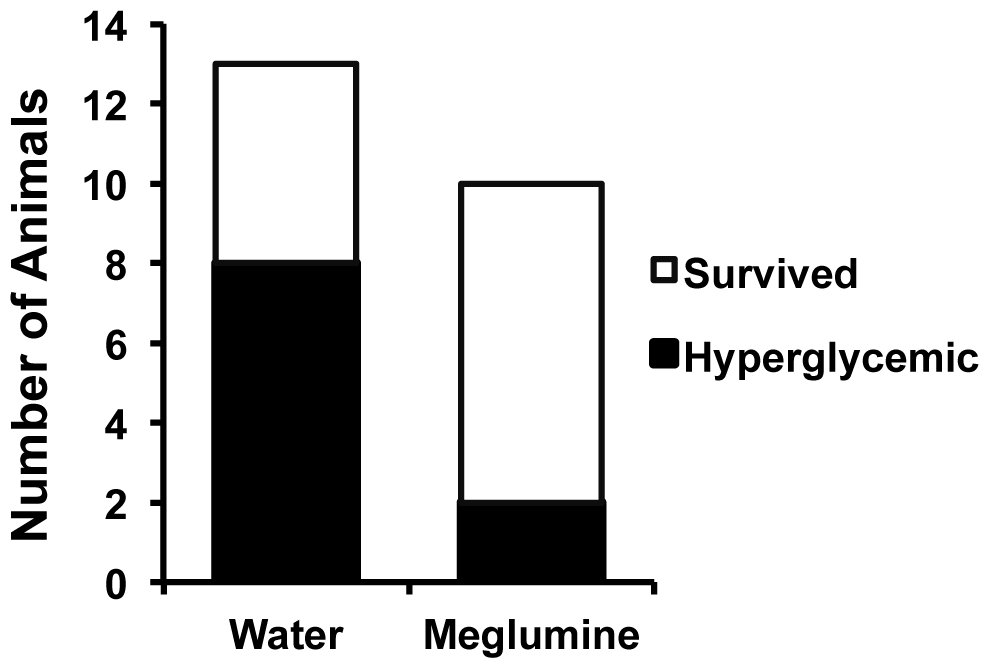
Meglumine reduces hyperglycemia. The figure presents the proportion of hyperglycemic mice that survived to the experimental endpoint of 7 months after receiving drinking water that was either untreated (**left bar**) or treated with meglumine (**right bar**).

**Figure 5:**
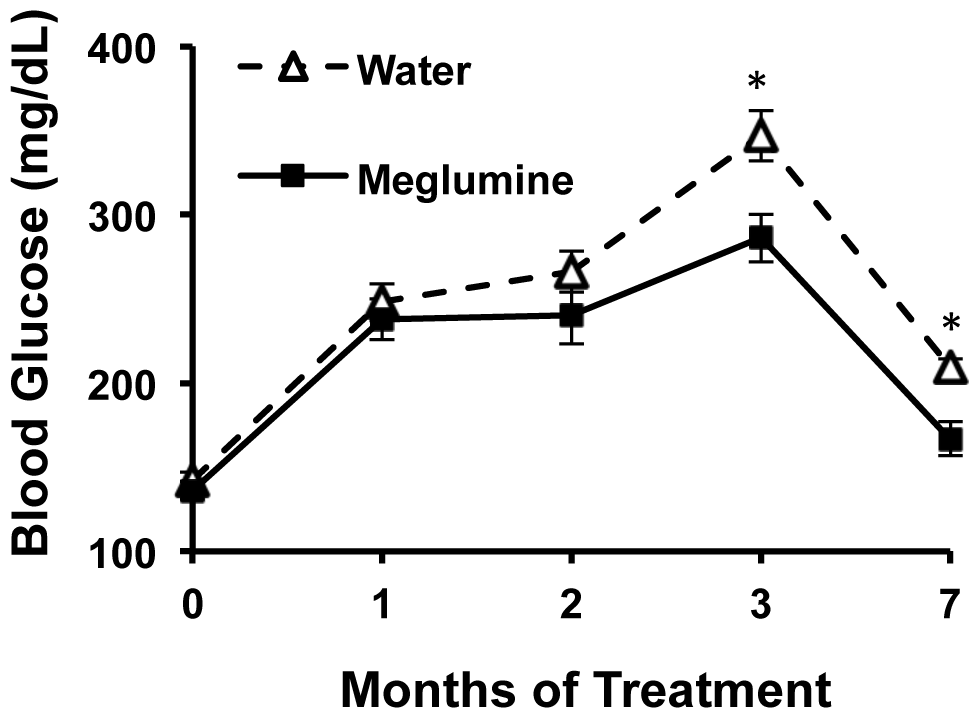
Meglumine reduces fasting blood glucose levels. KK.Cg-Ay/J mice treated with meglumine exhibited better glucose control and overall lower glucose levels than untreated mice. Most untreated hyperglycemic mice died between 3–7 months of age. Values are mean ± SE shown in the error bar. n = 20 for both groups (although not all time-points included measurements from all animals in each group). Asterisk designates P<0.05.

**Figure 6:**
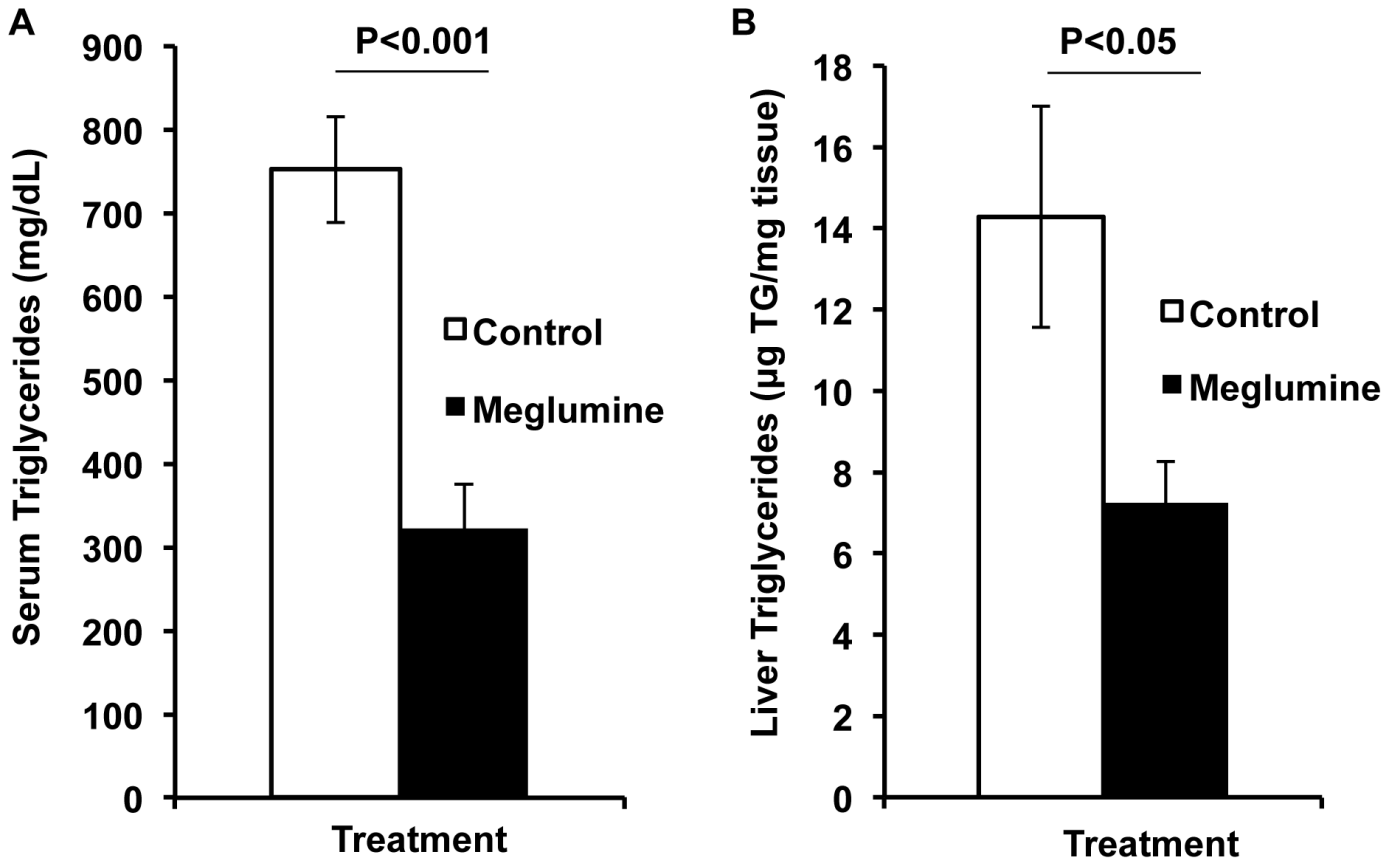
Serum and liver triglycerides are lowered by meglumine treatment. Seven month old KK.Cg-Ay/J mice were analyzed in this experiment having received drinking water that was untreated or treated with meglumine. A**:** Serum triglyceride levels. In untreated mice, levels were ∼750–800 mg/dL (**left bar, white**) whereas meglumine-treated mice displayed significantly lower levels of ∼300 mg/dL (**right bar, black**). Values are mean ± SE shown in the error bar. n = 10 for both groups. P = 0.00007. B**:** Liver triglyceride levels. Values are mean ± SE shown in the error bar. Water group, n = 10; meglumine group, n = 9. P = 0.043.

Mice treated with meglumine did not avoid the drinking water containing meglumine and their fluid consumption was similar to the untreated group during the course of the trial (Fig. S1). Notably, after two months of treatment, meglumine-treated mice performed better in a glucose tolerance test (Fig. 3), indicating that their glucose management was superior to control mice. Consistent with this result, meglumine-treated animals were less likely to exhibit hyperglycemia compared to their untreated peers (Fig. 4) and their average fasting levels of glucose were significantly lower (Fig. 5). In a coordinate manner, meglumine also significantly lowered triglyceride levels in both the liver and blood serum when compared with untreated mice (Fig. 6). Overall, these results supported the conclusion that meglumine may exert protective effects against the primary pathogenic drivers in diabetes and metabolic syndrome, perhaps including fatty liver disease.

### Meglumine Ameliorates Diabetic Nephropathy

**Figure 7:**
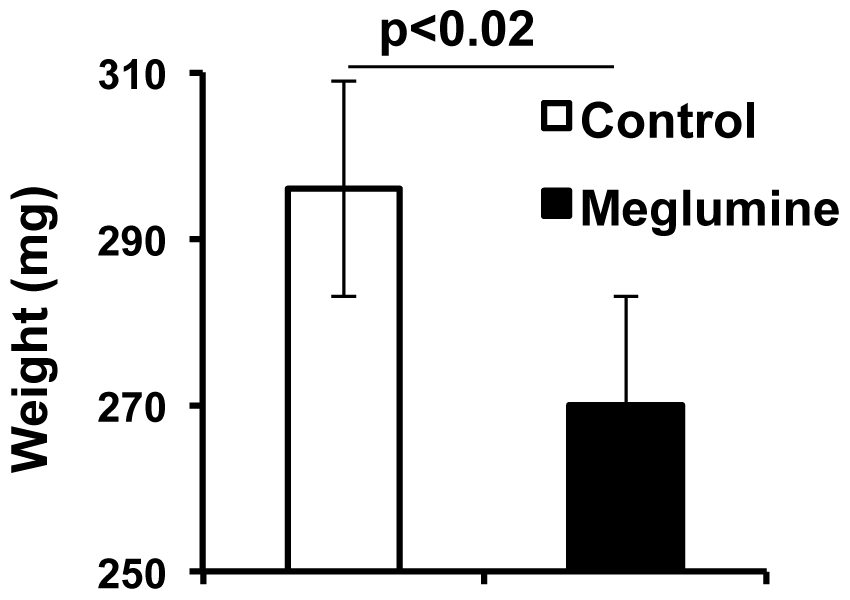
Kidney weight in diabetic animals is reduced by meglumine treatment. Kidney weights were measured postmortem in KK.Cg-Ay/J mice euthanized 7 months after initiation of the experiment. Untreated mice display higher kidney weights averaging ∼290 mg (**left bar, white**) whereas meglumine-treated mice display lower kidney weights averaging ∼260 mg (**right bar, black**). Values are mean ± SE shown in the error bar. n = 20 in both groups. P = 0.017.

KK.Cg-Ay/J mice quickly develop nephropathy and proteinuria, which is particularly acute in females, compared to other diabetic murine models [16]. The renal pathology displayed includes expansion and thickening of the mesangial matrix, Bowman’s capsule and tubules; proteinuria; and an increase in the albumin/creatinine ratio. In support of its beneficial effects on glycemic control and hyperlipidemia, we observed a significantly lower kidney weight in animals treated 7 months with meglumine. Kidneys from treated mice weighed an average of 297+/−SD mg compared to an average of 270+/−SD mg in the untreated control group (Fig. 7). This difference was mainly in female mice, where examination of a small number of subjects indicated a higher albumin/creatinine ratio in urine consistent with kidney pathology (data not shown). While both treated and untreated groups showed high levels of proteinuria, the levels were markedly higher in the untreated group. In further support of these observations, a qualitative ranking of the severity of kidney pathology showed a trend in the ability of meglumine to ameliorate nephritis, hydronephrosis, atrophy-fibrosis and glomerulosclerosis in female mice (Table 1). Taken together, these results furthered the evidence that meglumine exerted protective effects against diabetes in retarding the severity of nephropathy associated with diabetes. Overall, we concluded that meglumine has a number of heretofore undescribed medicinal properties that should be assessed clinically as a simple and possibly effective treatment for diabetes, fatty liver disease, muscle fatigue or metabolic syndrome.

**Table 1:** Trend in amelioration of nephropathy by meglumine in female diabetic mice.

	Nephritis	Hydronephrosis	Atrophy -Fibrosis	Glomerulosclerosis	Tubular Dilation
♂ Water (n = 3)	0.50±0.0	2.33±1.15	0.0±0.0	0.83±1.04	0.33±0.29
♂ Meg (n = 3)	0.50±0.0	3.00±0.0	1.00±0.0	0.75±0.35	2.00±0.0
♀ Water (n = 7)	1.14±0.63	0.57±0.79	0.64±0.48	0.79±0.57	1.43±0.53
♀ Meg (n = 6)	0.75±0.76	0.17±0.41	0.17±0.26	0.50±0.0	1.58±0.92

Kidney sections obtained from KK.Cg-Ay/J mice treated with or without meglumine in their drinking water for seven months were examined blindly by a kidney pathologist for evidence of nephritis, hydronephrosis, atrophy-fibrosis, glomerulosclerosis and tubular dilation. Severity was ranked qualitatively using a 0–4 scale where 0 = normal and 4 = severe lesion. The values presented in the Table represent the mean ± SE using this scale. Females in this model exhibit more nephropathy than males, the latter of which did not exhibit the beneficial trend seen in females. ♂, male; ♀, female; n, number of mice examined.

## Discussion

Insulin, as well as other oral and injectable diabetes medications combined with an exercise program, are the most common therapies prescribed to reduce blood glucose levels in diabetic patients. The mechanism by which glucose uptake occurs in skeletal muscle is well-established in the case of insulin, but there remain some gaps in knowledge concerning how skeletal muscle contraction leads to glucose uptake. Recent work has suggested that the AMPK-related kinase SNARK is a critical mediator of skeletal muscle contraction-stimulated glucose uptake [6]. While sorbitol can upregulate SNARK, its acute gastrointestinal side-effects make it unsuitable for routine treatment of diabetic patients. In this report, we documented the benefits of the sorbitol derivative meglumine as a potentially superior tool to improve glycemic control and muscle stamina and to delay the onset of diabetic complications such as nephropathy. Meglumine is regarded as safe by the U.S. FDA in concentrations as high as 100 mg/kg per day. It is commonly used as an excipient agent for drug formulations and the production of pharmaceutical grade material is inexpensive at less than US $300 per kg. Thus, the known safety and low cost render the potential medicinal properties of meglumine appealing to translate immediately to clinical testing for treatment of metabolic syndrome, fatty liver disease and diabetes, either independently or as an adjuvant supplement to exercise. Indeed, given the observed positive effects of meglumine on muscle stamina in mice, it is conceivable that this compound may offer a novel supplement to safely enhance strength or energy, and combat muscle fatigue in healthful or disease settings.

Compared to its structural relative sorbitol, we have shown meglumine to be a more potent stimulant of the AMPK-related kinase SNARK, which is implicated in mediating muscle contraction-induced glucose uptake [6]. In vivo tests revealed that oral meglumine, administered at 18 mM in drinking water freely consumed by animals in the present work, was safe, having no observable side-effects such as the diarrhea caused by sorbitol. Striking improvements in muscle stamina and weight control were observed in normal mice and improved glycemic control with a two-fold reduction in triglyceride levels in blood and liver was documented in highly diabetic mice. Kidney damage was the primary complication in the type II diabetes model we examined, and meglumine significantly reduced the observed severity of kidney damage in the model. Further work is required to understand the relationship between these observations. SNARK is a complex modifier of metabolic stress responses whose roles in normal physiology and pathophysiology are poorly understood. SNARK deficiency in mice is associated with increased body mass, serum triglycerides, hyperglycemia and glucose intolerance [7], but also increased physical activity and reduced inflammatory markers [21]. In humans, SNARK levels have been reported to be elevated in diabetic patients [22]. Our results warrant additional study of how meglumine improves muscle stamina, elicits cytoprotection in kidney of hyperglycemic rodents and improves glucose regulation. Indeed, since <20% of an administered oral dose of meglumine is absorbed into the bloodstream of rodents or dogs [13], further studies are warranted. For a better understanding of its apparent beneficial effects, observed in the present studies, primary actions of this compound at the level of gastrointestinal physiology and microbiome ecology should also be considered.

On the basis of its ability in rats to reduce levels of 3DG, an important driver of AGE accumulation in diabetes-associated complications and aging, meglumine has been conceptualized as an inhibitor of fructosamine-3-kinase (FN3K), the enzyme responsible for generating the chemical precursors of 3DG (A.T., F.K., A.M., unpublished data). *In vitro* studies also show that meglumine can ameliorate oxidative stress in a 3DG-rich environment, similar to what occurs in diabetic skin ulcers [15]. Our findings do not rule out such a role for meglumine, which could conceivably contribute to the reduced nephropathy observed in meglumine-treated animals. In a related vein, we do not know whether the ability of meglumine to affect glycemic control and hyperlipidemia actually reflects a direct effect on muscle. Even if this is the case, it is possible these benefits may be distally related, for example, by a direct impact on physical activity in the animal (i.e. by stimulating exercise). In future investigations, it will be important to assess the causal relations of meglumine on the response of SNARK, muscle function and metabolic parameters in diabetes, along with the precise molecular mechanisms that explain its effects on muscle or other tissues.

## Supporting Information

Figure S1 **Meglumine administration in drinking water does not affect daily fluid intake.** KK.Cg-Ay/J mice treated with meglumine (**dashed line**) showed no difference in daily fluid intake when compared to the untreated KK.Cg-Ay/J mice (**solid line**). Values are mean ± SE shown in the error bar. Both groups n = 20.
(TIFF)
